# Analysis of Maneuvering Targets with Complex Motions by Two-Dimensional Product Modified Lv’s Distribution for Quadratic Frequency Modulation Signals

**DOI:** 10.3390/s17061460

**Published:** 2017-06-21

**Authors:** Fulong Jing, Shuhong Jiao, Changbo Hou, Weijian Si, Yu Wang

**Affiliations:** College of Information and Communication Engineering, Harbin Engineering University, Harbin 150001, China; jing_fl@hrbeu.edu.cn (F.J.); jiaoshuhong@hrbeu.edu.cn (S.J.); swj0418@263.net (W.S.); wangyu1256@hrbeu.edu.cn (Y.W.)

**Keywords:** quadratic frequency modulation (QFM) signal, parameter estimation, multi-scale, multicomponent, Two-Dimensional product modified Lv’s distribution (2D-PMLVD)

## Abstract

For targets with complex motion, such as ships fluctuating with oceanic waves and high maneuvering airplanes, azimuth echo signals can be modeled as multicomponent quadratic frequency modulation (QFM) signals after migration compensation and phase adjustment. For the QFM signal model, the chirp rate (CR) and the quadratic chirp rate (QCR) are two important physical quantities, which need to be estimated. For multicomponent QFM signals, the cross terms create a challenge for detection, which needs to be addressed. In this paper, by employing a novel multi-scale parametric symmetric self-correlation function (PSSF) and modified scaled Fourier transform (mSFT), an effective parameter estimation algorithm is proposed—referred to as the Two-Dimensional product modified Lv’s distribution (2D-PMLVD)—for QFM signals. The 2D-PMLVD is simple and can be easily implemented by using fast Fourier transform (FFT) and complex multiplication. These measures are analyzed in the paper, including the principle, the cross term, anti-noise performance, and computational complexity. Compared to the other three representative methods, the 2D-PMLVD can achieve better anti-noise performance. The 2D-PMLVD, which is free of searching and has no identifiability problems, is more suitable for multicomponent situations. Through several simulations and analyses, the effectiveness of the proposed estimation algorithm is verified.

## 1. Introduction

In the past decades, radar signal processing has been a very important research field [1,2,3], especially estimating radar parameters [4,5,6]. The parameters of radar signals mainly include frequency [7], phase [8], and direction-of-arrival [9,10,11]. In this paper, we only focus on research into the frequency of the quadratic frequency modulation (QFM) signal [2,4]. Recently, many researchers have paid much attention to presenting effective inverse synthetic aperture radar (ISAR) imaging algorithms, which are important for high-resolution ISAR imaging and recognition of moving targets [12,13]. In general, the primary steps for ISAR imaging are range alignment [14,15] and phase adjustment [16,17], and translational-induced phase error correction. Then, the conventional range-Doppler (RD) method is utilized to reconstruct a well-focused ISAR image. However, in practical application, as the target’s motions are not smooth, the conventional RD method is not valid. For slow maneuvering targets, the azimuth echoes can be modeled as a linear frequency modulated (LFM) signal [18,19,20,21], and as the Doppler frequency shifts, we cannot obtain high-resolution imaging using the conventional RD. The chirp rate is identified as the cause of the target image defocus. Recently, because of this situation, many methods have been proposed, such as the fractional Fourier Transform (FrFT) [18], the stretch keystone-Wigner transform [19], the LPP-Hough transform [20], and the Lv’s distribution (LVD) [21]. However, for targets with complex motion, such as high maneuvering airplanes and ships fluctuating with oceanic waves, the azimuth echoes can be modeled as QFM signals, and methods for LFM signals are not suited for QFM signals. For ISAR imaging based on QFM signals, the chirp rate and the quadratic chirp rate induces a Doppler frequency shift and deteriorates the ISAR image.

Many algorithms about the parameter estimation of QFM signals for targets with complex motion have been proposed, and they generally fall into two categories: noncorrelation algorithms and correlation algorithms. Noncorrelation algorithms include the maximum likelihood algorithm [22], the quantification-based method [23], and the discrete chirp Fourier transform [24]. Correlation algorithms include the higher order ambiguity function (HAF) [2], the product higher order ambiguity function [25], the cubic phase function (CPF) [26,27], the product generalized cubic phase function [28], the product high-order matched phase transform (PHMT) [29], the HAF-integrated CPF (HAF-ICPF) [30], the scaled Fourier transform (SCFT)-based algorithm [31], the chirp rate-quadratic chirp rate distribution (CRQCRD) [32], the Modified Lv’s Distribution (MLVD) [33], and the new scaled Fourier transform (SCFT)-based algorithm [34]. Although noncorrelation algorithms can get high anti-noise performance with a lower signal-to-noise ratio (SNR) and have no cross-influence, they are not suitable for the QFM signal due to the high computation cost $\left(O\left(M^{2}N\log_{2}N\right)\right)$ [22,23,24], where N is the effective length of the slow time in ISAR, and M is the number of searching points, which is always greater than N. Thus, noncorrelation algorithms are worse in practical application. Compared to noncorrelation algorithms, although correlation algorithms are influenced by cross terms and have lower anti-noise performances, they have lower computational complexity and have advantages in radar system complexity. Thus, correlation algorithms are more practical than noncorrelation algorithms.

Through analyses of implementation, cross terms, error propagation, anti-noise performance and computational cost, the HAF-ICPF [30], the MLVD [33] and the algorithm presented in [34] will outperform other correlation algorithms [2,22,23,24,25,26,27,28,29,30,31,32,33,34]. Therefore, these algorithms are chosen to compare with the proposed estimation algorithm. However, some disadvantages still exist in these algorithms, as follows: (1) Due to requiring one-dimensional brute-force searching, the computation cost of the HAF-ICPF algorithm is still high. In addition, error propagation exists in HAF-ICPF, because it estimates the chirp rate and the quadratic chirp rate sequentially; (2) The algorithm presented in [34] overcomes brute-force searching and error propagation, but the algorithm does not have a high anti-noise performance, as redundancy information is not used; (3) Although the MLVD improves anti-noise performance by using redundancy information, it is still poor. In addition, the MLVD suffers from the identifiability problem when dealing with multiple components’ QFM signals with the same cubic order phase coefficients or the same cubic and quadratic order phase coefficients.

In this paper, according to the analyses above and the MLVD for the QFM signal, a novel algorithm, which is called 2D-PMLVD, is presented. The proposed estimation algorithm is based on a novel multi-scale parametric symmetric self-correlation function (PSSF) and the idea of keystone transform. By means of this processing, the QFM signal is transformed into different 2-D frequency domains, but at the same coordinate position. By multiplying all 2-D frequency domains, the proposed algorithm not only suppresses cross terms, but also improves anti-noise performance. Through analyses of the implementation, the 2D-PMLVD inherits advantages of the MLVD. Due to there being no brute-force searching, and its being implemented only using the FFT, the IFFT, and complex multiplication, the proposed estimation algorithm is easily implemented with low computation cost. Therefore, the 2D-PMLVD is more suitable in practical application.

The remainder of the paper is organized as follows. Section 2 introduces the model of QFM signals, and the principle of 2D-PMLVD for mono-QFM signals and multi-QFM signals. In addition, the performance of the 2D-PMLVD for multicomponent QFM signals is analyzed. Section 3 presents the details of the implementation in the discrete-time form. The anti-noise performance and the computational cost of the 2D-PMLVD are analyzed in Section 4. A conclusion is given in Section 5.

## 2. 2D-PMLVD with QFM Signal

In this section, the model of the QFM signals and the principle of the 2D-PMLVD are introduced, respectively.

For ISAR imaging of targets with complex motions, the geometry used here is based on the model in [31,32]. In this paper, range alignment and translational induced phase error correction, which can be referred to in [14,15,16,17], will not be discussed in detail. We will only focus on the processing of the Doppler frequency shift. Suppose that the radar transmits the LFM signal. According to the analyses in [31,32], after the range alignment and the translational induced phase error correction, azimuth echoes of the lth range cell take the form of multicomponent QFM signals

(1) $$s_{l}\left(t\right)=\sum_{p=1}^{P}A_{p}\exp\left[j2\pi \left(\phi_{1,p}t+\frac{1}{2}\phi_{2,p}t^{2}+\frac{1}{6}\phi_{3,p}t^{3}\right)\right]+z\left(t\right)$$

where P is the phase of the pth component, t denotes the slow time and its sampling frequency is the same as the pulse repetition frequency (PRF), z(t) denotes the additive complex white Gaussian noise with a variance of $\delta^{2}$, $\phi_{1,p}$, $\phi_{2,p}$, $\phi_{3,p}$ denote the centroid frequency, the chirp rate and the quadratic chirp rate, respectively.

### 2.1. 2D-PMLVD with Mono-QFM Signal

In order to facilitate the understanding of the algorithm, we first consider a noise-free QFM signal. It is represented as

(2) $$s\left(t\right)=A\exp\left[j\phi \left(t\right)\right]=A\exp\left[j2\pi \left(\phi_{1}t+\frac{1}{2}\phi_{2}t^{2}+\frac{1}{6}\phi_{3}t^{3}\right)\right]$$

where ϕ(t) is the phase function $\phi_{1}$, $\phi_{2}$ and $\phi_{3}$ denote the centroid frequency, the chirp rate and the quadratic chirp rate, respectively.

According to analyses in [21,33], a novel multi-scale PSSF is defined as

(3) $$R_{s}\left(t,\tau ,D\right)=s\left(t+\frac{\tau +a}{2}+D\right)s\left(t-\frac{\tau +a}{2}-D\right)s^{*}\left(t+\frac{\tau +a}{2}-D\right)s^{*}\left(t-\frac{\tau +a}{2}+D\right)$$

where a denotes a constant time-delay, τ denotes the lag time variable, and * denotes the complex conjugation. $D={\left\{d_{i}\right\}}_{i=1}^{L}(L>1)$ is the set of scale factors, and the selection criteria will be presented in Section 2.3.1. According to [33,34], the scale factor $d_{i}$ and the corresponding lag-time τ are utilized to complete the order reduction. Besides that, $d_{i}$ can reduce the number of self-correlations, which benefits the anti-noise performance.

Substituting (2) into (3) yields

(4) $$R_{s}\left(t,\tau ,D\right)=A^{4}\exp\left\{j2\pi \left[2\phi_{2}D\left(\tau +a\right)+2\phi_{3}Dt\left(\tau +a\right)\right]\right\}$$

It is easily seen from Equation (4) that the time variable t and lag variable τ couple with each other in exponential phase terms. For every scale factor, Equation (4) suffers the same influence of the coupling; we only need to analyze Equation (4) with one scale factor to explain the influence. Thus, Equation (4) is represented as

(5) $$R_{s}\left(t,\tau ,d_{i}\right)=A^{4}\exp\left\{j2\pi \left[2\phi_{2}d_{i}\left(\tau +a\right)+2\phi_{3}d_{i}t\left(\tau +a\right)\right]\right\}$$

The form of Equation (5) is the same as the self-correlation function defined for the MLVD [33] and LVD [21]. According to [21,33], we have known that the coupling influence energy accumulation. In order to analyze the influence of the coupling for the energy accumulation of the proposed algorithm, we use the energy accumulation method presented in [21,33], referred to as EA. The EA method is also the basic energy accumulation method used for the proposed algorithm. The EA method is divided into two steps. Firstly, perform FFT on Equation (5) with respect to t. Secondly, perform FFT on the first step with respect to τ. After the first step, the signal energy will peak along the inclined line $f_{t}=2\phi_{3}d_{i}\left(\tau +a\right)$. It is easily seen that the signal energy accumulation cannot be accomplished by the second step. Thus, in order to effectively accumulate the energy of the signal, the coupling needs to be removed. Here, the idea of keystone transformation is borrowed to rescale the time axis, which is defined as

(6) $$Κ\left(\Phi \left(t,\tau \right)\right)\to \Phi \left(\frac{t_{n}}{h\left(\tau +a\right)},\tau \right)$$

where a is a constant time-delay and h is a scaling factor different from $d_{i}$. The choice of parameters a and h will be presented in Section 2.3.1. Φ(t,τ) is the phase function with respect to (t,τ). Κ is the scaling operator. $t_{n}$ is denotes scaled time.

Applying the scaling operator Κ to (5), we have

(7) $$\begin{array}{ll} K\left[R_{x}\left(t,\tau ,d_{i}\right)\right] & =A^{4}\exp\left\{j2\pi \left[2\phi_{2}d_{i}\left(\tau +a\right)+\frac{2\phi_{3}d_{i}}{h}t_{n}\right]\right\}\\ & =A^{4}\exp\left\{j4\pi \phi_{2}d_{i}a\right\}\exp\left[j2\pi \left(2\phi_{2}d_{i}\tau +\frac{2\phi_{3}d_{i}}{h}t_{n}\right)\right] \end{array}$$

It is easily seen from (7) that the coupling has been removed by introducing the new time variable $t_{n}$. Then, by applying the first step of EA to Equation (7), the signal energy becomes a beeline $f_{t_{n}}=2\phi_{3}d_{i}/h$. Thus, the signal energy can be accumulated by the second step. The process can be expressed as

(8) $$\begin{array}{ll} L_{x}\left(f_{t_{n},i},f_{\tau ,i},d_{i}\right) & =FFT_{\tau }\left(FFT_{t_{n}}\left(Κ\left[R_{s}\left(t,\tau ,d_{i}\right)\right]\right)\right)\\ & =A^{4}\exp\left(j4\pi \phi_{2}ad_{i}\right)\delta \left(f_{\tau ,i}-2\phi_{2}d_{i}\right)\delta \left(f_{t_{n},i}-2\phi_{3}d_{i}/h\right) \end{array}$$

where δ(·) denotes the Dirac delta function, FFT(·) is the Fast Fourier transform operator. Equation (8) is similar to MLVD [33]. It can be easily seen from Equation (8) that a sole peak appears on the 2-D frequency domain $f_{\tau }-f_{t_{n}}$. The coordinate of the peak is $\left(f_{\tau ,i}^{\prime }=2\phi_{2}d_{i},f_{t_{n},i}^{\prime }=2\phi_{3}d_{i}/h\right)$. According to the coordinate of the peak, the parameters $\phi_{1}$, $\phi_{2}$, and $\phi_{3}$ can be estimated.

However when the EA method is applied to the multi-scale PSSF, the process is represented as

(9) $$L_{x}\left(f_{t_{n}},f_{\tau },D\right)={ℱ}_{\tau }\left({ℱ}_{t_{n}}\left(Κ\left[R_{s}\left(t,\tau ,D\right)\right]\right)\right)=A^{4}\exp\left(j4\pi \phi_{2}aD\right)\delta \left(f_{\tau }-2\phi_{2}D\right)\delta \left(f_{t_{n}}-\frac{2\phi_{3}D}{h}\right)$$

It is easily seen from Equation (9) that the mono-component signal will be translated into multi-peaks on $f_{\tau }-f_{t_{n}}$. The number of peaks is the same as the number of scale factors. Suppose that $d_{1}$ is the reference factor, and the peak which is correspond to $d_{1}$ is the reference peak. The relationships between reference peak and other peaks are represented as

(10) $$P_{1,i}=\frac{f_{\tau ,1}^{\prime }}{f_{\tau ,i}^{\prime }}=\frac{f_{t_{n},1}^{\prime }}{f_{t_{n},i}^{\prime }}=\frac{d_{1}}{d_{i}}\left(1\leq i\leq L\right)$$

where $P_{1,i}$ is zoom factor. $f_{\tau ,1}^{\prime }$ and $f_{t_{n},1}^{\prime }$ are the coordinates of the reference peak on the 2-D frequency domain. $f_{\tau ,i}^{\prime }$ and $f_{t_{n},i}^{\prime }$ are the coordinates of the peak which corresponds to $d_{i}$. It can be easily seen from Equation (10) that scale factor influences the location of the peak. Due to the values of scale factors have been selected by the selection criteria, which will be presented in Section 2.3.2, the relationships in Equation (10) can be easily obtained. According to these relationships, an improved energy accumulation method is presented, which is called $mSFT_{t,i}-FFT_{\tau ,i}$. The $mSFT_{t,i}-FFT_{\tau ,i}$ not only inherits advantages of decoupling but also improves the performance of energy accumulation. By using the $mSFT_{t,i}-FFT_{\tau ,i}$, the peaks of the QFM signal are transformed into different 2-D frequency domains, but at the same position under different scale factors. After multiplying all the 2-D frequency domains, a higher peak will appear. The $mSFT_{t,i}-FFT_{\tau ,i}$ will be presented in detail in Section 3. The processing is named 2D-PMLVD, which is represented as

(11) $$Q_{2D-PMLVD}=\prod_{i=1}^{L}FFT_{\tau ,i}\left(mSFT_{t,i}\left[R_{s}\left(t,\tau ,d_{i}\right)\right]\right)$$

2D-PMLVD not only suppresses cross terms, but also improves the anti-noise ability, which will be proved in Section 2.2 and Section 4.1.

2D-PMLVD is divided into four steps. Firstly, select one of the scale factors as the reference one and calculate the relationships between reference factor and other factors by Equation (10). Secondly, transform the QFM signal into different 2-D frequency domains based on $mSFT_{t,i}-FFT_{\tau ,i}$. Thirdly, multiply all the 2-D frequency domain to obtain the higher peak. Fourthly, detect the peak value to estimate the signal parameters.

For example, suppose that the set of scales contains two factors which are represented as $D=\left\{d_{1},d_{2}\right\}$. According to Equation (10), we get

(12) $$P_{1,2}=d_{1}/d_{2}$$

where $d_{1}$ is the reference scale. After applying $mSFT_{t,i}-FFT_{\tau ,i}$ to the QFM signal, two peaks will appear on different 2-D frequency domains. The reference peak will appear on $f_{\tau ,1}-f_{t_{n},1}$, and the other peak will appear on $f_{\tau ,2}-f_{t_{n},2}$. However, due to the $mSFT_{t,i}-FFT_{\tau ,i}$ operator, the relation between the different frequency domain is represented as

(13) $$f_{\tau ,2}-f_{t_{n},2}=\left(f_{\tau ,1}-f_{t_{n},1}\right)/P_{1,2}$$

**Figure 1:**
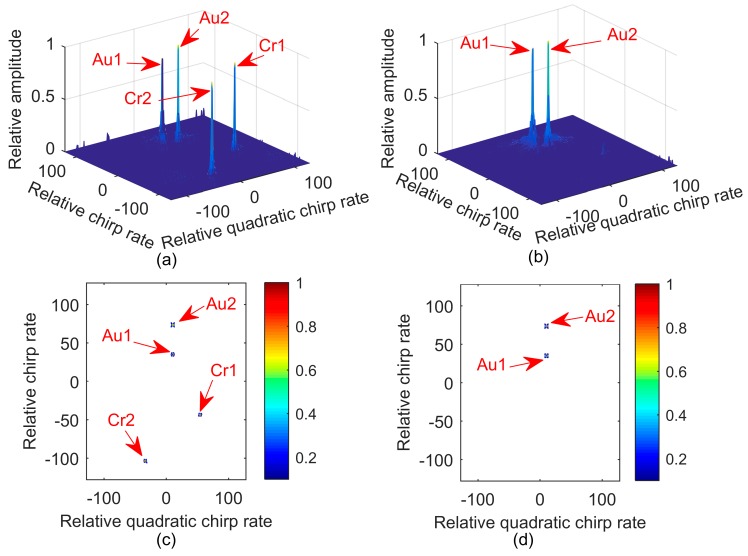
Simulation of Example 1. (**a**) Stereogram with MLVD; (**b**) Stereogram with 2D-PMLVD; (**c**) Contour with MLVD; (**d**) Contour with 2D-PMLVD.

**Figure 2:**
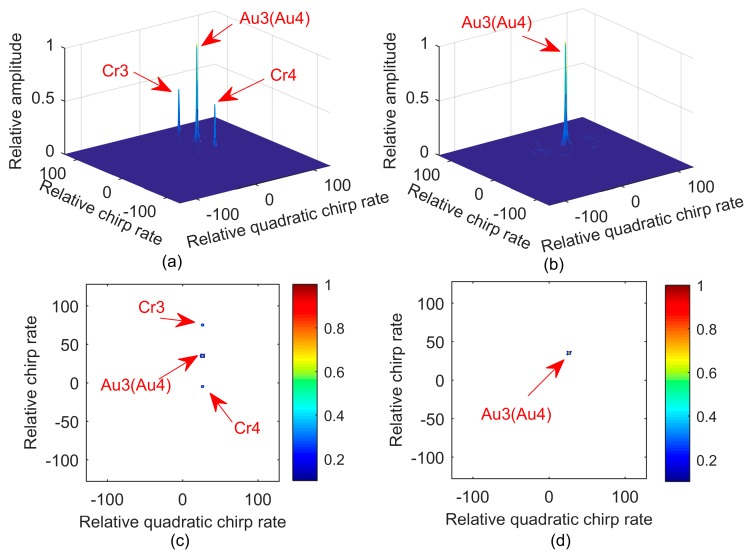
Simulation of Example 2. (**a**) Stereogram with MLVD; (**b**) Stereogram with 2D-PMLVD; (**c**) Contour with MLVD; (**d**) Contour with 2D-PMLVD.

In order words, the new frequency interval becomes $\left[0,1/P_{1,2}\right]$. Thus, because of the transformation, the two peaks appear at the same position. Then, a higher peak is obtained by multiplying the different frequency domains. According to the analysis in [25], the proposed algorithm can be improved when the number of scale factors is increased. Then, the computational cost will increase simultaneously. Hence, the number of scale factors will not be too large. In this paper, the number is selected as 2. By this selection, the cross terms can be effectively suppressed which are shown in Figure 1 and Figure 2.

### 2.2. 2D-PMLVD with Multi-QFM Signals

In this part, 2D-PMLVD with multi-QFM signals is analyzed. The proposed algorithm not only improves the ability to suppress cross terms, but also improves the performance of the EA method. The EA method, which is presented in MLVD [33] and LVD [21], has been proved to be effective for suppressing cross terms. Now, model the noise-free multi-QFM signals as

(14) $$s_{multi}\left(t\right)=\sum_{p=1}^{P}A_{p}\exp\left[j\phi \left(t\right)\right]=\sum_{p=1}^{P}A_{p}\exp\left[j2\pi \left(\phi_{1,p}t+\frac{1}{2}\phi_{2,p}t^{2}+\frac{1}{6}\phi_{3,p}t^{3}\right)\right]$$

To formulate the cross term problem arising from multi-QFM signals, we first consider two signals (P=2) in Equation (14), which is represented as

(15) $$\begin{array}{ll} s_{multi}\left(t\right) & =\sum_{p=1}^{2}A_{p}\exp\left[j\phi \left(t\right)\right]\\ & =A_{1}\exp\left[j2\pi \left(\phi_{1,1}t+\frac{1}{2}\phi_{2,1}t^{2}+\frac{1}{6}\phi_{3,1}t^{3}\right)\right]+A_{2}\exp\left[j2\pi \left(\phi_{1,2}t+\frac{1}{2}\phi_{2,2}t^{2}+\frac{1}{6}\phi_{3,2}t^{3}\right)\right] \end{array}$$

For ease of understanding, the multi-scale PSSF can be written as

(16a) $$R_{s}\left(t,\tau ,D\right)=R\left(t+\frac{\tau +a}{2},D\right)R^{*}\left(t-\frac{\tau +a}{2},D\right)$$

where

(16b) $$R\left(t,D\right)=s\left(t+D\right)s^{*}\left(t-D\right)$$

Submitting (14) into (16b), we obtain

(17) $$R\left(t,D\right)=R_{auto}\left(t,D\right)+R_{cross}\left(t,D\right)$$

where $R_{auto}\left(t,D\right)$ and $R_{cross}\left(t,D\right)$ denote auto-terms and cross terms with different scale factors, respectively. In order to describe the shortcoming of the EA method and explain the principle of the energy accumulation method in the proposed algorithm, we firstly consider one scale factor in Equation (17). It is represented as

(18a) $$R\left(t,d_{i}\right)=R_{auto}\left(t,d_{i}\right)+R_{cross}\left(t,d_{i}\right)$$

Submitting (14) into (18a),

(18b) $$\begin{array}{ll} R_{auto}\left(t,d_{i}\right) & =A_{1}^{2}\exp\left[j2\pi \left(2\phi_{1,1}d_{i}+\frac{1}{3}\phi_{3,1}d_{i}^{3}\right)\right]\times \exp\left[j2\pi \left(2\phi_{2,1}d_{i}t+\phi_{3,1}d_{i}t^{2}\right)\right]\\ & +A_{2}^{2}\exp\left[j2\pi \left(2\phi_{1,2}d_{i}+\frac{1}{3}\phi_{3,2}d_{i}^{3}\right)\right]\times \exp\left[j2\pi \left(2\phi_{2,2}d_{i}t+\phi_{3,2}d_{i}t^{2}\right)\right] \end{array},$$

(18c) $$\begin{array}{l} R_{cross}\left(t,d_{i}\right)=A_{1}A_{2}\exp\left[j2\pi \left(\phi_{1,1}d_{i}+\phi_{1,2}d_{i}+\frac{1}{2}\phi_{2,1}d_{i}^{2}-\frac{1}{2}\phi_{2,2}d_{i}^{2}+\frac{1}{6}\phi_{3,1}d_{i}^{3}+\frac{1}{6}\phi_{3,2}d_{i}^{3}\right)\right]\\ \times \exp\left\{j2\pi \left[\left(\phi_{1,1}-\phi_{1,2}+\phi_{2,1}d_{i}+\phi_{2,2}d_{i}+\frac{1}{2}\phi_{3,1}d_{i}^{2}-\frac{1}{2}\phi_{3,2}d_{i}^{2}\right)t+\frac{1}{2}\left(\phi_{2,1}-\phi_{2,2}+\phi_{3,1}d_{i}+\phi_{3,2}d_{i}\right)t^{2}+\frac{1}{6}\left(\phi_{3,1}-\phi_{3,2}\right)t^{3}\right]\right\}\\ +A_{1}A_{2}\exp\left\{j2\pi \left[\phi_{1,1}d_{i}+\phi_{1,2}d_{i}+\frac{1}{2}d_{i}^{2}\left(\phi_{2,2}-\phi_{2,1}\right)+\frac{1}{6}d_{i}^{3}\left(\phi_{3,1}+\phi_{3,2}\right)\right]\right\}\\ \times \exp\left\{j2\pi \left[\left(\phi_{1,2}-\phi_{1,1}+\phi_{2,1}d_{i}+\phi_{2,2}d_{i}+\frac{1}{2}\phi_{3,2}d_{i}^{2}-\frac{1}{2}\phi_{3,1}d_{i}^{2}\right)t+\frac{1}{2}\left(\phi_{2,2}-\phi_{2,1}+\phi_{3,1}d_{i}+\phi_{3,2}d_{i}\right)t^{2}+\frac{1}{6}\left(\phi_{3,2}-\phi_{3,1}\right)t^{3}\right]\right\} \end{array}.$$

According to [21], Equation (16a) is similar to the form of the self-correlation function defined for the LVD. Thus, under multi-QFM signals, if Equation (18c) takes the form of the LFM signal, the cross terms will accumulate as an auto-term. Let $\Delta \phi_{1}=\phi_{1,1}-\phi_{1,2}$, $\Delta \phi_{2}=\phi_{2,1}-\phi_{2,2}$, and $\Delta \phi_{3}=\phi_{3,1}-\phi_{3,2}$. LFM signals will exist in the cross terms as in the following two cases [32,35]:

1. $\Delta \phi_{1}\neq 0$, $\Delta \phi_{2}\neq 0$, and $\Delta \phi_{3}=0$.
2. $\Delta \phi_{1}\neq 0$, $\Delta \phi_{2}=0$, and $\Delta \phi_{3}=0$.

In these two cases, the spurious peaks will appear on $f_{\tau }-f_{t_{n}}$, which greatly interferes with the performance of the detection. However, 2D-PMLVD overcomes the problem by introducing the multi-scale PSSF and $mSFT_{t,i}-FFT_{\tau ,i}$. This will be analyzed in detail as follows.

#### 2.2.1. Case One

When $\Delta \phi_{1}\neq 0$, $\Delta \phi_{2}\neq 0$, and $\Delta \phi_{3}=0$, the $R_{cross}\left(t,d_{i}\right)$ is represented as

(19) $$\begin{array}{ll} R_{cross}\left(t,d_{i}\right) & =A_{1}A_{2}\exp\left\{j2\pi \left[\phi_{1,1}d_{i}+\phi_{1,2}d_{i}+\frac{1}{2}d_{i}^{2}\left(\phi_{2,1}-\phi_{2,2}\right)+\frac{1}{3}\phi_{3,:}d_{i}^{3}\right]\right\}\\ & \times \exp\left\{j2\pi \left[\left(\phi_{1,1}-\phi_{1,2}+\phi_{2,1}d_{i}+\phi_{2,2}d_{i}\right)t+\frac{1}{2}\left(\phi_{2,1}-\phi_{2,2}+2\phi_{3,:}d_{i}\right)t^{2}\right]\right\}\\ & +A_{1}A_{2}\exp\left\{j2\pi \left[\phi_{1,1}d_{i}+\phi_{1,2}d_{i}+\frac{1}{2}d_{i}^{2}\left(\phi_{2,2}-\phi_{2,1}\right)+\frac{1}{3}\phi_{3,:}d_{i}^{3}\right]\right\}\\ & \times \exp\left\{j2\pi \left[\left(\phi_{1,2}-\phi_{1,1}+\phi_{2,1}d_{i}+\phi_{2,2}d_{i}\right)t+\frac{1}{2}\left(\phi_{2,2}-\phi_{2,1}+2\phi_{3,:}d_{i}\right)t^{2}\right]\right\} \end{array}$$

where $\phi_{3,:}=\phi_{3,1}=\phi_{3,2}$. It can be easily seen from Equation (19) that $R_{cross}\left(t,d_{i}\right)$ takes the form of two LFM signals. This means that the cross terms will accumulate two spurious peaks. The coordinates of the spurious peaks are

(20) $$(f_{\tau ,i}^{Cr1}=\phi_{1,1}-\phi_{1,2}+\phi_{2,1}d_{i}+\phi_{2,2}d_{i},f_{t_{n},i}^{Cr1}=\phi_{2,1}-\phi_{2,2}+2\phi_{3,:}d_{i})\;\mathrm{and}\;\;\left(f_{\tau ,i}^{Cr2}=\phi_{1,2}-\phi_{1,1}+\phi_{2,1}d_{i}+\phi_{2,2}d_{i},f_{t_{n},i}^{Cr2}=\phi_{2,2}-\phi_{2,1}+2\phi_{3,:}d_{i}\right)$$

It can be easily seen from Equation (18b) that $R_{auto}\left(t,d_{i}\right)$ takes the form of two LFM signals. The coordinates of the true peaks are

(21) $$\left(f_{\tau ,i}^{Au1}=2\phi_{2,1}d_{i},f_{t_{n},i}^{Au1}=2\phi_{3,1}d_{i}\right)\;\mathrm{and}\;\left(f_{\tau ,i}^{Au2}=2\phi_{2,2}d_{i},f_{t_{n},i}^{Au2}=2\phi_{3,2}d_{i}\right)$$

For different scale factors, the coordinates of the true peaks meet Equation (10), and can be represented as

(22) $$P_{i,j}=\frac{f_{\tau ,i}^{Au1}}{f_{\tau ,j}^{Au1}}=\frac{f_{t_{n},i}^{Au1}}{f_{t_{n},j}^{Au1}}=\frac{f_{\tau ,i}^{Au2}}{f_{\tau ,j}^{Au2}}=\frac{f_{t_{n},i}^{Au2}}{f_{t_{n},j}^{Au2}}$$

However, through the analysis of Equation (20), the coordinates of the spurious peaks do not meet Equation (10). In other words, the coordinates are not proportional to the scale factor. Thus, the spurious peaks cannot be transformed into the same position by the operator $mSFT_{t,i}-FFT_{\tau ,i}$. After multiplying all the frequency domain, the spurious peaks will not be enhanced. Compared with the true peaks, the spurious peaks are “suppressed”. The result is based on the premise that the scale factors are different. This is because if the scale factors are equivalent, the spurious peaks are in the same position, which means that the cross terms cannot be suppressed. This is proved by Example 1. The selection criterion for the scale factor is introduced in Section 2.3.2.

**Example** **1.** *We consider two QFM signals* P1 *and* P2. *Signal parameters are set as follows: $A_{1}=1$, $\phi_{1,1}=89$, $\phi_{2,1}=42$, $\phi_{3,1}=4$ for P1; and $A_{2}=1$, $\phi_{1,2}=10$, $\phi_{2,2}=20$, $\phi_{3,2}=4$ for P2. The sampling frequency $F_{s}$ is set to 256 Hz. The effective signal length* N *is equal to 256. Both* a *and* h *are set to one. The quadratic chirp rate of* P1 *and* P2 *are equal to each other. Figure 1a shows the results of MLVD with a scale factor equal to 56.* Au1 *and* Au2 *denote the true peaks of the auto-terms. Because $\phi_{3,2}=\phi_{3,1}$, two spurious peaks, denoted by* Cr1 *and Cr2, exist in the 2-D frequency domain alongside the true peaks. It can be easily seen that the spurious peaks are even higher than the true ones in Figure 1a. This creates a serious challenge for signal detection. Figure 1b shows the results of 2D-PMLVD with two scale factors equal to 56 and 64. Because the multi-scale PSSF and the operator* $mSFT_{t_{n},i}-FFT_{\tau ,i}$ *were applied, the spurious peaks of the cross terms are suppressed, and the true peaks of the auto-terms are greatly strengthened. Figure 1c,d gives the contour of Figure 1a,b, respectively.*

#### 2.2.2. Case Two

When $\Delta \phi_{1}\neq 0$, $\Delta \phi_{2}=0$, and $\Delta \phi_{3}=0$, $R_{auto}\left(t,d_{i}\right)$ is represented as

(23) $$\begin{array}{ll} R_{auto}(t,d_{i})= & \left\{A_{1}^{2}\exp\left[j2\pi \left(2\phi_{1,1}d_{i}+\frac{1}{3}\phi_{3,:}d_{i}^{3}\right)\right]+A_{2}^{2}\exp\left[j2\pi \left(2\phi_{1,2}d_{i}+\frac{1}{3}\phi_{3,:}d_{i}^{3}\right)\right]\right\}\\ & \times \exp\left[j2\pi \left(2\phi_{2,:}d_{i}t+\phi_{3,:}d_{i}t^{2}\right)\right] \end{array}$$

where $\phi_{2,:}=\phi_{2,1}=\phi_{2,2}$. And $R_{cross}\left(t,d_{i}\right)$ is represented as

(24) $$\begin{array}{ll} R_{cross}\left(t,d_{i}\right) & =A_{1}A_{2}\exp\left[j2\pi \left(\phi_{1,1}d_{i}+\phi_{1,2}d_{i}+\frac{1}{3}\phi_{3,:}d_{i}^{3}\right)\right]\times \exp\left\{j2\pi \left[\left(\phi_{1,1}-\phi_{1,2}+2\phi_{2,:}d_{i}\right)t+\phi_{3,:}d_{i}t^{2}\right]\right\}\\ & +A_{1}A_{2}\exp\left[j2\pi \left(\phi_{1,1}d_{i}+\phi_{1,2}d_{i}+\frac{1}{3}d_{i}^{3}\phi_{3,:}\right)\right]\times \exp\left\{j2\pi \left[\left(\phi_{1,2}-\phi_{1,1}+2\phi_{2,:}d_{i}\right)t+\phi_{3,:}d_{i}t^{2}\right]\right\} \end{array}$$

Unlike case one, $R_{auto}\left(t,d_{i}\right)$ only takes the form of one LFM signal in case two. That is because the two LFM signals are the same. It means that, by the proposed algorithm, only a higher peak of the auto-terms is obtained. The coordinates of the true peak are $\left(2\phi_{2,:}d_{i},2\phi_{3,:}d_{i}\right)$. It can be easily seen that the coordinates are proportional to the scale factor.

It can be easily seen from Equation (24) that $R_{cross}\left(t,d_{i}\right)$ also takes the form of two LFM signals. The coordinates of the two peaks are $\left(\phi_{1,2}-\phi_{1,1}+2\phi_{2,:}d_{i},2\phi_{3,:}d_{i}\right)$ and $\left(\phi_{1,1}-\phi_{1,2}+2\phi_{2,:}d_{i},2\phi_{3,:}d_{i}\right)$, respectively. Thus, the coordinates are not proportional to the scale factor. This is the same as in case one.

The process for suppressing the cross terms is the same as in case one. After applying 2D-PMLVD to case two, the spurious peaks can effectively be suppressed and the identifiability is improved. This is shown in Example 2.

**Example** **2.** *We consider two QFM signals* P3 *and P4. Signals parameters are set as follows: $A_{3}=1$, $\phi_{1,3}=10$, $\phi_{2,3}=20$, $\phi_{3,3}=10$ for P3; and $A_{4}=1$, $\phi_{1,4}=30$, $\phi_{2,4}=20$, $\phi_{3,4}=10$ for P4. The sampling frequency* $F_{s}$ *is set to 256 Hz. The effective signal length* N *is equal to 256. Both* a *and* h *are set to one. The quadratic chirp rate and chirp rate of* P3 *and* P4 *are the same. Figure 2a shows the results of MLVD with a scale factor equal to 56. Because the quadratic chirp rate and chirp rate are the same, the auto-term is accumulated into one peak, which is represented as Au3(Au4) . Cr3 and* Cr4 *denote the spurious peaks. Figure 2b shows the results of 2D-PMLVD with two scale factors. By means of 2D-PMLVD, the spurious peaks of the cross terms are suppressed, and the true peak is greatly strengthened. Figure 2c,d gives the contour of Figure 2a,b, respectively.*

### 2.3. Parameter Selection and Resolution

In this part, the selection criteria of the parameters a, h and scale factors are presented. In practical applications, the QFM signal is of finite-length and discrete. This means that the selection criteria only need to be discussed in a finite-length discrete setting.

#### 2.3.1. Selection of a and h

We know that the optimal value of ah is equal to one [21,36]. Define the discrete parameter pair (a,h) as $\left(m/f_{s},h\right)$. In order to directly read the quadratic chirp rate from the 2-D frequency domain, the optimal value of h is set to one. According to the length of redundancy, the selection of the parameters follows two criteria.

*Criterion 1*: According to the analysis in [21], as long as the signal is long enough to guarantee at least 1 s redundancy, that is to say $N_{all}/f_{s}-N^{2D-PMLVD}/f_{s}\geq 1$, the parameters are

(25) $$\left(m=f_{s},\;h=1\right)$$

$N_{all}$ and $N^{2D-PMLVD}$ denote the length of the signal and the effective length of the signal, respectively. In this case, the parameters of a and h are equal to one, and the previously processed data can be used as redundancy information to perform the proposed algorithm. Because 2D-PMLVD includes a scale factor set that is different from the LVD, the effective length of 2D-PMLVD is shorter than that of LVD. It means that 2D-PMLVD is much more suited to fulfilling the condition of redundancy.

*Criterion 2*: However, in practical applications, the total sampling time may be very short, even less than 1 s, or the frequencies of the chirp signal may vary quickly with time even if the length of signal is long enough. The parameter selection criterion is not satisfied by the above conditions. The new condition is $N_{all}/f_{s}-N^{2D-PMLVD}/f_{s}<1$. To guarantee the condition that a is equal to one, the parameters are represented as

(26) $$\left(m=N_{all}-N^{2D-PMLVD},\;h=f_{s}/\left(N_{all}-N^{2D-PMLVD}\right)\right)$$

The derivation of Equation (26) is in accordance with [21], which aims at making h as close as possible to one under the condition of ah=1.

#### 2.3.2. Selection of $d_{i}$

According to the analysis in [33], we know that the scale factors not only reduce order, but also benefit anti-noise performance. Thus, their selection is very important for 2D-PMLVD. According to the analysis in [37], we know that the optimal *d_i_* value is obtained by minimizing the values for the MSE of chirp rate and quadratic chirp rate. This is represented as

(27) $$d_{i}\approx \left\lceil 0.089N_{all}\right\rceil$$

where ⌈•⌉ denotes the round up operator. We have seen in Section 2.2 that, in order to suppress the cross terms, the scale factors in D will be different. When we move away from the optimal choice, we will certainly obtain a sub-optimal set. As far as accuracy is concerned, it would be better to have the effective signal length be as close to the optimal length as possible, which suggests that the selection of the other scale factors in the set D should be close to $d_{i}^{opt}$.

For a mono-QFM signal, the values of scale factors may be equivalent, as there is no cross term. However, in actual processing, we do not know the QFM signal is mono-component or multi-component. Thus, the scale factors in D are different.

#### 2.3.3. Resolution

In the proposed algorithm, which is similar to the frequency resolution of Fourier transform, the resolution for representing the parameters of CR and QCR relate to the signal length, which makes sense, since the 1-D QFM signals are converted into the 2-D single frequency signals in the CR-QCR domain. In this paper, due to the sampling scheme of Claasen and Mecklenbräuker (CM) and the finite-length effect, the resolution for representing the parameters of CR and QCR are different. Moreover, the resolutions also relate to the scale factors. We have seen that the multi-scale PSSF can be divided into Equation (16a,b), and that Equation (16a) is the same as PSIAF, which is presented in [21]. Thus, some theorems in [21] can also be applied to this paper. Applying Equation (16b) to a QFM signal, the auto-terms can be represented as different LFM signals with different scale factors and the centroid frequency and the chirp rate of the ith LFM signal can be expressed as

(28) $$f_{i}=2d_{i}\varphi_{2,p}\;\mathrm{and}\;\gamma_{i}=2d_{i}\varphi_{3,p}$$

Suppose Δ denotes sampling interval. According to the Appendix B in reference [21], we have known that the resolutions of centroid frequency and the chirp rate are expressed as

(29) $$\delta f_{i}=1/N_{i}\Delta \;\mathrm{and}\;\delta \gamma_{i}=2/\left(hN_{i}\Delta \right)$$

where $N_{i}$ denotes the length of the ith LFM signal; and $N_{i}$ is represented as $N_{i}=N_{all}-2d_{i}$, where $N_{all}$ denotes the length of the QFM signal. According to Equations (28) and (29), the corresponding resolution for $\varphi_{2,p}$ and $\varphi_{3,p}$ is represented as

(30) $$\delta \varphi_{2,p}=1/\left(2d_{i}N_{i}\Delta \right)\;\mathrm{and}\;\delta \varphi_{3,p}=1/\left(hd_{i}N_{i}\Delta \right)$$

It can be easily seen from Equation (30) that different scale factors correspond to different resolutions; and the resolutions are influenced by the worst resolutions in the proposed algorithm. Thus, the resolutions are influenced not only by the signal length but also by the scale factors.

## 3. Implementation

In this section, the discrete implementation of 2D-PMLVD will be discussed by using the sampling scheme of Claasen and Mecklenbräuker (CM) [38]. The discrete form of (4) expressed as

(31) $$R_{s}\left(t,\tau ,D\right)=A^{4}\exp\left\{j2\pi \left[2\phi_{2}D\left(2mT_{s}+a\right)+2\phi_{3}DnT_{s}\left(2mT_{s}+a\right)\right]\right\}$$

The key step of the implementation is the scaling transform. Similar to the keystone transform, the operator Κ can be fulfilled by using DFT-IFFT or SFT-IFFT, which are analyzed in detail in [19]. According to the analysis in [19], the SFT-IFFT transformation only uses complex multiplications and FFT based on the scaling principle, and is more suitable for implementation than DFT-IFFT. If the Fourier transform is represented as

(32) S(f)=ℱ(r(t))=∫r(t)exp(−j2πft)dt

then the representation of SFT is expressed as

(33) $$S\left(\lambda ,f\right)=\bar{ℱ}\left(r\left(t\right)\right)=\int r\left(t\right)\exp\left(-j2\pi \lambda ft\right)dt$$

where $\lambda =\left(2mT_{s}+a\right)h/N$.

According to the analysis in Section 2.1 and Section 2.2, the energy of the auto-terms is expected to be accumulated into the same position under different scale factors. However, the EA method, which is presented in [21,33], is only valid for mono-scale factors, and is invalid for multi-scale factors. According to the analysis of SFT, an improved energy accumulation method is proposed, called modified SFT-FFT $\left(mSFT_{t,i}-FFT_{\tau ,i}\right)$. The operator is divided into two parts: a performing $mSFT_{t,i}\left[•\right]$ operator along the t axis and a performing $FFT_{\tau ,i}\left[•\right]$ operator along the τ axis.

The $mSFT_{t,i}\left[•\right]$ is different from Equation (33), which is represented as

(34) $$mSFT_{t,i}\left[R\left(t,\tau ,d_{i}\right)\right]=\int R\left(t,\tau ,d_{i}\right)\exp\left(-j2\pi \lambda f_{i}^{\Delta }t\right)dt$$

where $R\left(t,\tau ,d_{i}\right)$ denotes the PSSF when scale factor is $d_{i}$. $f_{i}^{\Delta }=f_{1}/P_{1,i}\left(1\leq i\leq L\right)$, where $f_{1}$ denotes the reference frequency domain and $f_{i}^{\Delta }$ denotes the ith frequency domain, which corresponds to the ith scale factor. Compared with the interval of $f_{1}$, the interval of $f_{i}^{\Delta }$ becomes $\left[0,1/P_{1,i}\right]$ by introducing $P_{1,i}$. The variable λ in $mSFT_{t,i}\left[•\right]$ is the same as in Equation (33). Thus, the coupling between t and τ can also be removed by the $mSFT_{t,i}\left[•\right]$ operator. Based on Equation (10), we know that $P_{1,i}$ denotes the relationships between reference energy peak position and the ith energy peak position. By using the zoom factor $P_{1,i}$, the $mSFT_{t,i}\left[•\right]$ operator changes the frequency interval. Then, under different scale factors, the signal energy of the auto-term is accumulated at the same position on the $f_{t_{n}}$ axis. In other words, under different scale factors, the signal energy of the auto-term is accumulated into the same beeline which is represented as $f_{t_{n}}=X\left(\tau \right)$, where X(τ) denotes the function of τ. Then, applying the $FFT_{\tau ,i}\left[•\right]$ operator along the τ axis, the signal energy of the auto-term is accumulated as a sharp peak in the 2-D frequency domain. For the ith scale factor, the number of FFT point is $Num_{1}/P_{1,i}$, where $Num_{1}$ denotes the number of the FFT point for the reference scale factor. Thus, $mSFT_{t,i}-FFT_{\tau ,i}$ is represented as

(35) $$mSFT_{t,i}-FFT_{\tau ,i}\left[R_{s}\left(t,\tau ,d_{i}\right)\right]=FFT_{\tau ,i}\left(mSFT_{t,i}\left[R_{s}\left(t,\tau ,d_{i}\right)\right]\right)$$

**Figure 3:**
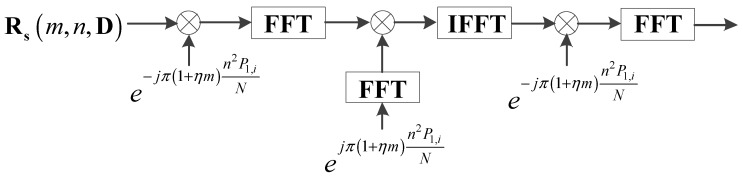
Flowchart of $mSFT_{t,i}$ $-FFT_{\tau ,i}$.

Through analyses of the implementation, $mSFT_{t,i}\left[•\right]$ not only inherits advantages of the SFT with regard to the transformation of the time-domain, but also improves the performance in the frequency-domain. Moreover, the computation cost does not increase too much. The flowchart of $mSFT_{t,i}-FFT_{\tau ,i}$ is shown in Figure 3.

The main steps of estimation parameters by 2D-PMLVD are illustrated as follows.

Input: QFM signals, D. The factors in D are obtained by the selection criterion introduced in Section 2.3.1.

Step1): Substitute QFM signals and D into Equation (3), and $R_{s}\left(t,\tau ,D\right)$ is obtained.

Step2):

**for** i=1:L **do**

Apply the $mSFT_{t,i}$ operator to $R_{s}\left(t,\tau ,d_{i}\right)$ along the t axis by Equation (34).

Apply the $FFT_{\tau ,i}\left[•\right]$ operator to Equation (34) along the τ axis, and we can obtain the data, represented as $\beta_{i}$.

**end for**

Step3): Multiply all $\beta_{i}$, we can obtain different peaks, which correspond to different QFM components.

Step4): Detect the peak value to estimate the signal parameter chirp rate and quadratic chirp rate.

Output: the QFM signal parameter chirp rate and quadratic chirp rate.

## 4. Analyses of Anti-Noise Performance and Computational Cost

In this section, the anti-noise performance and computational cost, which play important roles in parameters estimation [32,33,34], will be analyzed for 2D-PMLVD. HAF-ICPF [30], MLVD [33], and the algorithm presented in [34] are chosen as comparisons.

### 4.1. Anti-Noise Performance Analysis

In this simulation, we consider a noisy QFM signal. According to analysis methods employed in other estimation algorithms [19,21,32,33,34,36,39], we utilize input-output SNR [21,32] and mean square error (MSE) [37] to evaluate the anti-noise performance of the proposed algorithm with Example 3.

**Figure 4:**
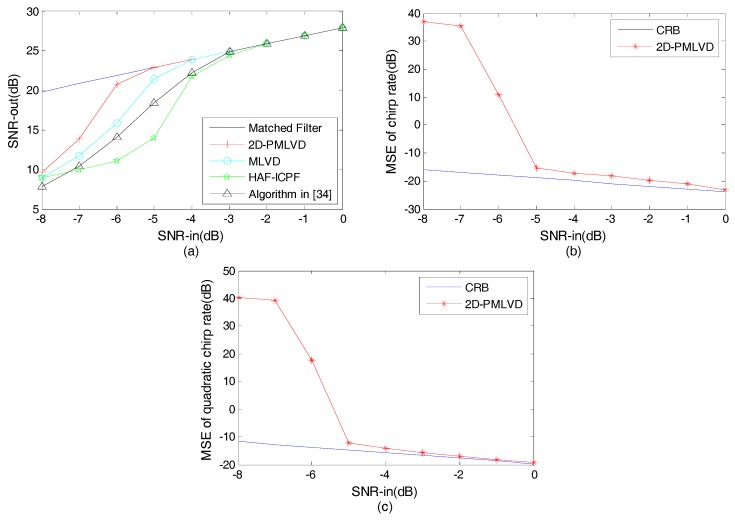
Anti-noise performance analysis. (**a**) Input-output SNR comparison; (**b**) MSE of the chirp rate estimation; (**c**) MSE of the quadratic chirp rate estimation.

**Example** **3.** *We consider one QFM signal denoted by P5. The parameters of* P5 *are the same as P1. The sampling frequency* $F_{s}$ *is set to 256 Hz. The effective signal length* N *is equal to 256. Both* a *and* h *are set to one. The scale factors are equal to 56 and 64. The input SNRs tested in Figure 4 are $SNR_{in}=\left[-8:1:0\right]$, and 100 trials are performed for each* $SNR_{in}$ *value.*

The input-output SNR comparison of the four algorithms is shown in Figure 4a, and the solid line denotes the result of the matched filter for P5. HAF-ICPF contains HAF and ICPF. HAF is used to reduce the order of the QFM signal, and then ICPF is used to accumulate the energy and estimate the parameter. We know that the threshold SNR of HAF is low [2]. However, according to [40], ICPF will improve the threshold SNR. Therefore, the threshold SNR of HAF-ICPF is −2dB. The evidence for this result can be obtained as described in [33,37]. The algorithm presented in [34], which employs a fourth-order autocorrelation function, is based on coherent accumulation [34]. The algorithm presented in [34] defines a novel parametric autocorrelation function to complete the order reduction and energy accumulation, which benefits anti-noise performance, although redundancy information is not utilized. Thus, in Figure 4a, the threshold SNR of the algorithm presented in [34] is −3 dB. In contrast to the algorithm presented in [34], MLVD utilizes redundancy information, and therefore the anti-noise performance is based on a better anti-noise algorithm. However, MLVD only utilizes one scale factor, which is not favorable for anti-noise performance. Thus, its threshold SNR is −4 dB, which conforms to the result in [33]. In Figure 4a, the threshold SNR of 2D-PMLVD is −5 dB, which is better than the other three algorithms. The advantages include: (1) the energy accumulation of 2D-PMLVD is coherent; (2) in the defined multi-scale PSSF, the redundancy information and multi-scale factors not only reduce the number of self-correlations, but also benefit anti-noise performance [33,37]; and (3) by the $mSFT_{t_{n},i}-FFT_{\tau ,i}$ and production, the cross terms are effectively suppressed. In addition, it is worthwhile noting that the error propagations exist in HAF-ICPF as a result of the sequential estimation of chirp rate and the quadratic chirp rate. However, this problem does not exist in the other three algorithms.

For 2D-PMLVD, the observed MSEs for the chirp rate and the quadratic chirp rate estimation are plotted in Figure 4b,c as functions of SNR. The corresponding Cramer-Rao bounds (CRBs), which can be obtained as described in [41,42], are also shown by the solid line in the Figure 4. Obviously, in Figure 4b,c, both the MSEs of the chirp rate and quadratic chirp rate estimations are close to CRB when SNR≥−5dB. Therefore, the threshold SNR of the 2D-PMVLD is −5 dB for MSEs, and these results conform to those in Figure 4a.

### 4.2. Computational Cost

The anti-noise performance of PMVLD has been analyzed in the Section 4.1. In this section, the computational cost will be analyzed for PMVLD.

For HAF-ICPF, the main implementation procedures include HAF (O(N)) [2] and ICPF $\left(O\left(N^{3}\right)\right)$ [40], where N denotes signal length. Thus, the HAF-ICPF computational cost is $\left(O\left(N^{3}\right)\right)$ [30,33,34]. For the algorithm presented in [34], its main implementation procedures include a parametric autocorrelation function $\left(O\left(N^{2}\right)\right)$, the GSCFT $\left(O\left(N^{2}\log_{2}N\right)\right)$ [34] and an FFT operation $\left(O\left(N^{2}\log_{2}N\right)\right)$. Thus, its computational cost is $\left(O\left(N^{2}\log_{2}N\right)\right)$ [34]. For MLVD, the main implementation procedures include the parametric symmetric self-correlation function $\left(O\left(N^{2}\right)\right)$, the keystone transform $\left(O\left(N^{2}\log_{2}N\right)\right)$ and the FFT operation $\left(O\left(N^{2}\log_{2}N\right)\right)$. Thus, the MLVD computational cost is also $\left(O\left(N^{2}\log_{2}N\right)\right)$ [33]. For 2D-PMLVD, the main implementation procedures include the defined parametric symmetric self-correlation function $\left(O\left(N^{2}\right)\right)$, the $mSFT_{t,i}-FFT_{\tau ,i}$ $\left(O\left(N^{2}\log_{2}N\right)\right)$, and (L−1) products of frequency domain. However, L is usually a small number (e.g., 2), meaning that the additional cost is not excessive. Thus, the computational cost of 2D-PMLVD is $\left(O\left(N^{2}\log_{2}N\right)\right)$. Table 1 lists the computational costs of these four estimation algorithms.

## 5. Conclusions

In this paper, we propose 2D-PMLVD for QFM signals by employing multi-scale PSSF and $mSFT_{t,i}-FFT_{\tau ,i}$. Due to the characteristics of multi-scale PSSF, 2D-PMLVD can utilize $mSFT_{t,i}-FFT_{\tau ,i}$ to remove coupling and accumulate energy to the same position under different scale factors. Based on the products of different 2-D frequency domains, cross terms are suppressed and auto-terms are enhanced. The main contributions of the proposed method include the following: (1) it eliminates the procedure of parameter searching; (2) it improves identifiability for multi-QFM signals; (3) it improves anti-noise performance; and (4) it achieves a balance between low computational cost and high performance with low SNR.

## Figures and Tables

**Table 1:** Computational Cost.

Estimation Algorithm	Computational Cost
HAF-ICPF	$\left(O\left(N^{3}\right)\right)$
The algorithm presented in [34]	$\left(O\left(N^{2}\log_{2}N\right)\right)$
MLVD	$\left(O\left(N^{2}\log_{2}N\right)\right)$
2D-PMLVD	$\left(O\left(N^{2}\log_{2}N\right)\right)$

## References

[1] Zhou C., Gu Y., He S., Shi Z. (2017). A robust and efficient algorithm for coprime array adaptive beamforming. IEEE Trans. Veh. Technol..

[2] Barbarossa S., Petrone V. (1997). Analysis of polynomial-phase signals by the integrated generalized ambiguity function. IEEE Trans. Signal Process..

[3] Gu Y., Leshem A. (2012). Robust adaptive beamforming based on interference covariance matrix reconstruction and steering vector estimation. IEEE Trans. Signal Process..

[4] Song Y.E., Wang C.G., Shi P. (2016). Algorithm based on the linear canonical transform for qfm signal parameters estimation. IET Signal Process..

[5] Gu Y., Goodman N.A. (2017). Information-theoretic compressive sensing kernel optimization and bayesian Cramér-Rao bound for time delay estimation. IEEE Trans. Signal Process..

[6] Shi Z., Zhou C., Gu Y., Goodman N.A., Qu F. (2017). Source estimation using coprime array: A sparse reconstruction perspective. IEEE Sens. J..

[7] Wang P., Li H., Djurovic I., Himed B. (2010). Performance of instantaneous frequency rate estimation using high-order phase function. IEEE Trans. Signal Process..

[8] Mo S., Wang Y., Liu C. (2015). An estimation algorithm for phase errors in synthetic aperture radar imagery. IEEE Geosci. Remote Sens. Lett..

[9] Wang B., Zhang Y.D., Wang W. (2017). Robust doa estimation in the presence of mis-calibrated sensors. IEEE Signal Process. Lett..

[10] Guo M., Tao C., Wang B. (2017). An improved doa estimation approach using coarray interpolation and matrix denoising. Sensors.

[11] Zhou Y., Zhang Y.D., Shi Z., Jin T., Wu X. (2017). Compressive Sensing Based Coprime Array Direction-of-Arrival Estimation. IET Commun..

[12] Berizzi F., Mese E.D., Diani M., Martorella M. (2001). High-resolution isar imaging of maneuvering targets by means of the range instantaneous doppler technique: Modeling and performance analysis. IEEE Trans. Image Process..

[13] Li Y., Xing M., Su J., Quan Y. (2013). A new algorithm of isar imaging for maneuvering targets with low snr. IEEE Trans. Aerosp. Electron. Syst..

[14] Zhu D., Li Y., Zhu Z. (2007). A keystone transform without interpolation for sar ground moving-target imaging. IEEE Geosci. Remote Sens. Lett..

[15] Xing M., Wu R., Bao Z. (2005). High resolutio isar imaging of high speed moving targets. IEE Proc. Radar Sonar Navig..

[16] Wahl D.E., Eichel P.H., Ghiglia D.C., Jakowatz C.V. (1994). Phase gradient autofocus—A robust tool for high resolution sar phase correction. IEEE Trans. Aerosp. Electron. Syst..

[17] Xi L., Liu G., Ni J. (1999). Autofocusing of isar images based on entropy minimization. IEEE Trans. Aerosp. Electron. Syst..

[18] Almeida L.B. (1994). The fractional fourier transform and time-frequency representations. IEEE Trans. Signal Process..

[19] Lv X., Xing M., Wan C., Zhang S. (2010). Isar imaging of maneuvering targets based on the range centroid doppler technique. IEEE Trans. Image Process..

[20] Bi G., Li X., Samson See C.M. (2011). Lfm signal detection using lpp-hough transform. Signal Process..

[21] Lv X., Bi G., Wan C., Xing M. (2011). Lv’s distribution: Principle, implementation, properties, and performance. IEEE Trans. Signal Process..

[22] Abatzoglou T.J. Fast maximum likelihood joint estimation of frequency and frequency rate. Proceedings of the IEEE International Conference on Acoustics, Speech, and Signal Processing, ICASSP ’86.

[23] Zheng J. (2013). Fast parameter estimation algorithm for cubic phase signal based on quantifying effects of doppler frequency shift. Prog. Electromagn. Res..

[24] Wu L., Wei X., Yang D., Wang H. (2012). Isar imaging of targets with complex motion based on discrete chirp fourier transform for cubic chirps. IEEE Trans. Geosci. Remote Sens..

[25] Barbarossa S., Scaglione A., Giannakis G.B. (1998). Product high-order ambiguity function for multicomponent polynomial-phase signal modeling. IEEE Trans. Signal Process..

[26] O’Shea P. (2002). A new technique for instantaneous frequency rate estimation. IEEE Signal Process. Lett..

[27] O’Shea P. (2004). A fast algorithm for estimating the parameters of a quadratic fm signal. IEEE Trans. Signal Process..

[28] Wang Y., Jiang Y. (2011). Inverse synthetic aperture radar imaging of maneuvering target based on the product generalized cubic phase function. IEEE Geosci. Remote Sens. Lett..

[29] Wang Y., Jiang Y. (2009). Isar imaging of a ship target using product high-order matched-phase transform. IEEE Geosci. Remote Sens. Lett..

[30] Wang Y. (2012). Inverse synthetic aperture radar imaging of manoeuvring target based on range-instantaneous- doppler and range-instantaneous-chirp-rate algorithms. IET Radar Sonar Navig..

[31] Bai X., Tao R., Wang Z., Wang Y. (2014). Isar imaging of a ship target based on parameter estimation of multicomponent quadratic frequency-modulated signals. IEEE Trans. Geosci. Remote Sens..

[32] Zheng J., Su T., Zhang L., Zhu W. (2014). Isar imaging of targets with complex motion based on the chirp rate—Quadratic chirp rate distribution. IEEE Trans. Geosci. Remote Sens..

[33] Li Y., Su T., Zheng J., He X. (2015). ISAR imaging of targets with complex motions based on modified lv’s distribution for cubic phase signal. IEEE J. Sel. Top. Appl. Earth Obs. Remote Sens..

[34] Zheng J., Liu H., Liao G., Su T. (2016). Isar imaging of targets with complex motions based on a noise-resistant parameter estimation algorithm without nonuniform axis. IEEE Sens. J..

[35] Zheng J., Su T., Zhu W., Zhang L. (2015). Isar imaging of nonuniformly rotating target based on a fast parameter estimation algorithm of cubic phase signal. IEEE Trans. Geosci. Remote Sens..

[36] Lv X., Xing M., Zhang S., Bao Z. (2009). Keystone transformation of the wigner-ville distribution for analysis of multicomponent lfm signals. Signal Process..

[37] Djurovic I., Simeunovic M., Djukanovic S., Wang P. (2012). A hybrid cpf-haf estimation of polynomial-phase signals: Detailed statistical analysis. IEEE Trans. Signal Process..

[38] Claasen T.A.C.M., Mecklenbräuker W.F.G. (1980). The wigner distribution—A tool for time-frequency signal analysis—Part II: Discrete time signals. Philips J. Res..

[39] Luo S., Bi G., Lv X., Hu F. (2013). Performance analysis on lv distribution and its applications. Digit. Signal Process..

[40] Wang P., Li H., Djurović I., Himed B. (2010). Integrated cubic phase function for linear fm signal analysis. IEEE Trans. Aerosp. Electron. Syst..

[41] Peleg S., Porat B. (1991). The cramer-rao lower bound for signals with constant amplitude and polynomial phase. IEEE Trans. Signal Process..

[42] Peleg S., Porat B. (1991). Linear fm signal parameter estimation from discrete-time observations. IEEE Trans. Aerosp. Electron. Syst..

